# Attitudes Toward and Use of Prostate-Specific Antigen Testing Among Urologists and General Practitioners in Germany: A Survey

**DOI:** 10.3389/fonc.2021.691197

**Published:** 2021-06-04

**Authors:** Sanny Kappen, Verena Jürgens, Michael H. Freitag, Alexander Winter

**Affiliations:** ^1^ Division of Epidemiology and Biometry, Department of Health Services Research, School of Medicine and Health Sciences, Carl von Ossietzky University Oldenburg, Oldenburg, Germany; ^2^ Division of General Practice, Department of Health Services Research, School of Medicine and Health Sciences, Carl von Ossietzky University Oldenburg, Oldenburg, Germany; ^3^ University Hospital for Urology, Klinikum Oldenburg, Department of Human Medicine, School of Medicine and Health Sciences, Carl von Ossietzky University Oldenburg, Oldenburg, Germany

**Keywords:** prostatic neoplasms, early detection of cancer, prostate-specific antigen, physicians, healthcare surveys, attitudes, guideline adherence

## Abstract

**Background:**

In 2020, around 1.4 million new prostate cancer (PCa) cases were recorded worldwide. Early detection of PCa by prostate-specific antigen (PSA) screening remains debated, leading to different specialist-specific recommendations in PCa guidelines. This study aimed to assess attitudes toward and use of PSA testing among urologists in Germany and general practitioners (GPs) in Lower Saxony (Germany).

**Methods:**

A nationwide questionnaire was sent to urologists *via* the mailing lists of the Professional Association of German Urologists and the German Urological Society. A version of the questionnaire for GPs was sent to email addresses *via* the Association of Statutory Health Insurance Physicians Lower Saxony. The online questionnaires covered use of PSA testing, information communication, handling of test results, and handling of/knowledge about national and international guidelines and recommendations on early detection of PCa. Statistical analysis was performed at a descriptive level.

**Results:**

In total, 432 of 6,568 urologists (6.6%) and 96 of 1,579 GPs (6.1%) participated in this survey. Urologists and GPs differed in their attitudes and approaches toward PSA testing. Most urologists (86.8%, n=375) judged the test as “very meaningful” or “meaningful”, compared with 52.1% (n=50) of GPs. Almost two-thirds of the urologists (64.4%, n=278) viewed the PCa mortality reduction by PSA testing as proven, compared with one-fifth of GPs (20.8%, n=20). Almost 80% of male urologists (79.9%, n=291) indicated that they would undergo a PSA test in the future (again), compared with 55.1% of male GPs (n=38). In addition, 56.3% (n=243) of urologists stated that “considerably more than half” or “almost all” men aged 45 years or older received a PSA test, compared with 19.8% (n=19) of GPs.

**Conclusions:**

Urologists are more convinced about the PSA test than GPs. PSA testing is therefore used more often in urological settings, although the preselected patient population must be considered. In accordance with specialist-specific recommendations, GPs show a more reserved approach toward PSA testing. Instead of focusing on different attitudes and recommendations on PSA testing, the exchange between specialist groups should be improved to achieve a consistent approach to PSA testing.

## Introduction

In 2020, around 1.4 million new prostate cancer (PCa) cases were recorded worldwide, accounting for 14% of all new cancer cases in men (1). PCa is the second most frequent cancer and the fifth leading cause of cancer death in men worldwide. However, PCa screening by PSA testing is still debated. Large-scale screening studies have shown inconsistent results for PSA with respect to a decrease in PCa mortality (2–8). A systematic review concluded that at best, PCa screening leads to a small reduction in disease-specific mortality over 10 years but has no effect on overall mortality (9). The net benefit of PSA testing for PCa screening remains unclear because of adverse effects (e.g., overdiagnosis and overtreatment), leading to different recommendations for PSA testing (10–13).

In healthcare systems featuring evidence-based care, physicians are expected to adhere to relevant clinical guidelines. A coordinated and harmonized approach (e.g., a European level strategy on the early detection of PCa) would be helpful for physicians. The European Union (EU) Cancer Plan recommends the European Commission supports an EU-wide PCa awareness campaign, even mandating and endorsing clinical guidelines on the early detection and diagnosis of PCa (14). Contributors to the EU Cancer Plan believe this will increase the number of well-informed men, ensure better quality of life outcomes for patients with PCa, reduce prostate-specific mortality, and decrease costs for publicly funded health systems.

In addition to guidelines, health insurance and individual patient- and physician-related factors influence the early detection and treatment of PCa (15–18). Because of the ambiguity related to the PSA test, the test is not part of the statutory early detection program in Germany. Instead, the PSA test is offered by many physicians as an individual health service that is self-paid by the patient. A patient’s decision to undergo or forgo PSA screening is influenced by their physician’s recommendation (19).

Several guidelines highlight the importance of an informed decision-making process (10–13). The way the patient is informed influences the use of PSA testing and the patient’s satisfaction with early detection (20, 21). Specialist-specific recommendations described in the German S3 guideline may explain the variation in daily practice of PSA testing between specialist groups (13, 18). The extent to which these specialist-specific recommendations affect physicians’ attitudes toward and use of PSA testing remains unclear.

The aim of this study was to describe the attitudes toward and use of PSA testing among urologists and GPs in Germany.

## Material and Methods

### Study Design, Setting, and Participants

In August/September 2019, a one-time online questionnaire was sent by email to all urologists that were members of the Professional Association of German Urologists or the German Urological Society. This allowed both urologists working in clinics and outpatient settings to be included. A version of the questionnaire for GPs was sent to members’ email addresses *via* the Association of Statutory Health Insurance Physicians Lower Saxony. Reminders were sent out to the physicians. No reimbursements were paid.

### Questionnaire Development and Data Collection

The two German questionnaires used in this study (one for urologists and one for GPs) were adapted from earlier questionnaires on PSA testing that were developed by our working group (18, 22). In general, the versions for urologists and GPs were similar. Despite the fact that the version for urologists was useable for urologists working in clinics, as well as for urologists working in outpatient care, some specific questions and/or answering categories were adapted to the specialization of the physicians (e.g., the answering category “directly refer the patient to a urologist” was just available for GPs). The two versions were developed using SoSci Survey software (www.soscisurvey.de). In addition to collecting demographic data and practice/clinic characteristics, the online questionnaires included questions addressing physician attitudes, clinical practice, and familiarity with recommendations related to PSA testing. Daily practice variation in PSA testing was also explored following presentation of three standardized case scenarios. Acceptance and ease of use of the questionnaires were tested for the earlier versions among selected urologists, GPs, and health scientists. The questionnaires are available on request.

### Statistical Analysis

Response proportions were calculated separately for urologists and GPs. Participant characteristics and survey responses relating to attitudes and approaches toward PSA testing and guideline use were analyzed at a descriptive level. In most cases, absolute and relative frequencies were used for categorical variables. Data analysis was performed using IBM SPSS Statistics version 25.

### Ethics

A positive ethics vote was obtained from the Medical Ethics Committee of the Carl von Ossietzky University Oldenburg (No. 2019/041).

## Results

### Response Proportions and Responder Characteristics

**Figure 1:**
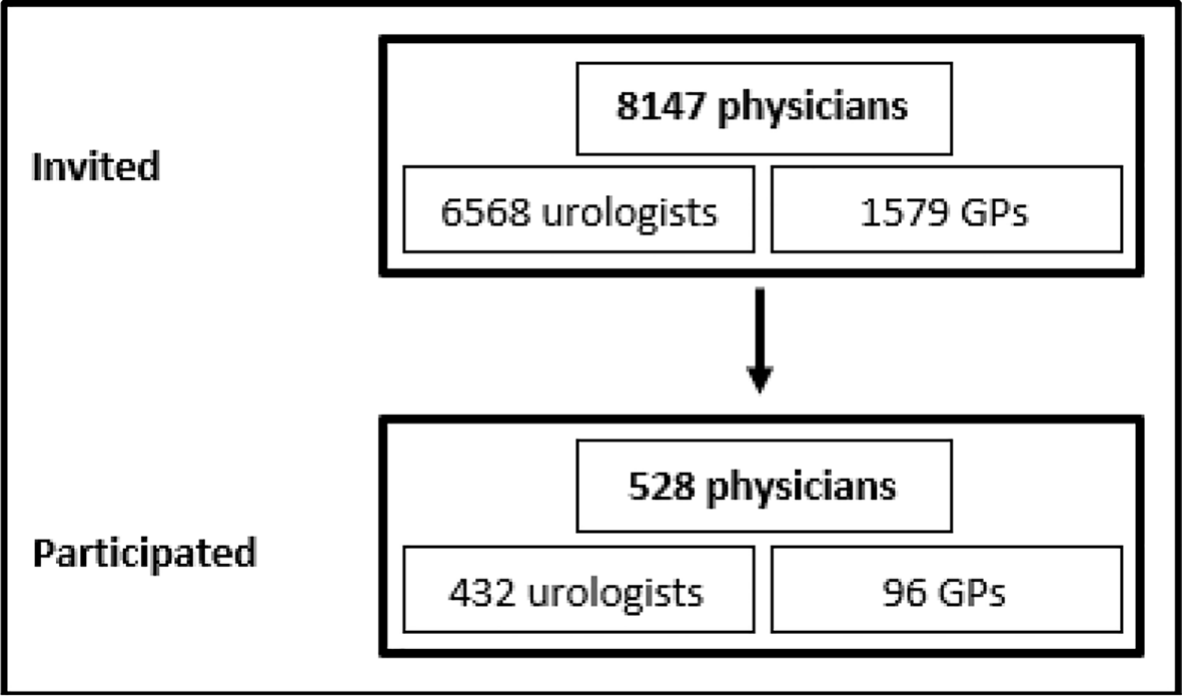
Flowchart of survey participants. GPs, general practitioners.

In total, 432 of the 6,568 contacted urologists (6.6%) and 96 of 1,579 GPs (6.1%) completed the online questionnaire correctly (**Figure 1**). Characteristics of the participating urologists and GPs are shown in **Table 1**. Most physicians were male and had 10 or more years of work experience since finishing their specialty (urologists: 72.5%, n=313; GPs: 67.8%, n=65). All age groups were represented. Most urologists worked in outpatient care (68.5%, n=296), 25.2% (n=109) worked in hospitals, and a few (3.5%, n=15) worked in other settings (e.g., the federal office of public health or as a court-appointed expert). Of the urologists, 13.2% (n=57) worked in Lower Saxony. GPs either had their own practice (39.6%, n=38) or worked in a group practice (54.2%, n=52). Almost half of the GPs (46.9%, n=45) had participated in a seminar on PSA testing after their medical studies, although in most cases (55.6%, n=25) this course was completed 1–5 years ago.

**Table 1:** Characteristics of urologists and general practitioners [n (%)].

Variable	Categories	Urologists (n=432)	GPs (n=96)
**Sex**	*Male*	364 (84.3)	69 (71.9)
	*Female*	68 (15.7)	27 (28.1)
	*Diverse*	0 (0.0)	0 (0.0)
**Age (in years)**	*≤ 29*	4 (0.9)	0 (0.0)
	*30-34*	25 (5.8)	2 (2.1)
	*35-39*	37 (8.6)	3 (3.1)
	*40-44*	47 (10.9)	13 (13.5)
	*45-49*	43 (10.0)	18 (18.8)
	*50-54*	94 (21.8)	17 (17.7)
	*55-59*	78 (18.1)	18 (18.8)
	*60-64*	67 (15.5)	10 (10.4)
	*≥ 65*	37 (8.6)	15 (15.6)
**Specialist**	*Yes*	400 (92.6)	89 (92.7)
	*No*	32 (7.4)	7 (7.3)
**Work experience (since finishing specialty, in years)**	*0-4*	42 (9.7)	4 (4.2)
	*5-9*	45 (10.4)	19 (19.8)
	*10-19*	127 (29.4)	30 (31.3)
	*≥ 20*	186 (43.1)	35 (36.5)
	*Missing replies*	32 (7.4)	8 (8.3)
**Main work setting**	*Outpatient care*	296 (68.5)	n/a
	*Hospital*	109 (25.2)	n/a
	*Other*	15 (3.5)	n/a
	*Missing replies*	12 (2.8)	n/a

GPs, general practitioners; n/a, not applicable; PSA, prostate-specific antigen.

### Influence and Awareness of Guidelines on PCa

For urologists, the national German S3 guideline was the favored guideline on PSA testing (**Table 2**). More than 80% of the urologists (83.1%, n=359) stated they knew this guideline in detail. GPs appeared to be aware of the German Society of General Practice and Family Medicine (DEGAM) recommendation and the German S3 guideline to a similar extent. Most GPs had heard about the guidelines but stated they did not know the entire content (DEGAM: 56.3%, n=54; S3: 60.4%, n=58). Most urologists had heard about major studies on PSA screening (European Randomized Study of Screening for Prostate Cancer [ERSPC]: 77.8%, n=336; Prostate, Lung, Colorectal, and Ovarian trial [PLCO]: 58.8%, n=254), whereas more than 60% of the GPs had never heard of these studies (ERSPC: 60.4%, n=58; PLCO: 66.7%, n=64).

**Table 2:** Awareness and influence of guidelines and recommendations on prostate cancer by urologists and general practitioners [n (%)].

Question	Categories	Urologists (n=432)				GPs (n=96)			
		*DEGAM recommendation*	*German S3 guideline*	*ERSPC*	*PLCO*	*DEGAM recommendation*	*German S3 guideline*	*ERSPC*	*PLCO*
**Are you aware of the following guidelines and study recommendations/results regarding PSA testing (irrespective of the version)?**	*No, I never heard about it*	162 (37.5)	3 (0.7)	53 (12.3)	135 (31.3)	5 (5.2)	11 (11.5)	58 (60.4)	64 (66.7)
	*Yes, I heard about it, but the content is not entirely known*	179 (41.4)	27 (6.3)	161 (37.3)	148 (34.3)	54 (56.3)	58 (60.4)	23 (24.0)	17 (17.7)
	*Yes, I know it in detail*	48 (11.1)	359 (83.1)	175 (40.5)	106 (24.5)	24 (25.0)	14 (14.6)	2 (2.1)	2 (2.1)
	*Missing replies*	43 (10.0)	43 (10.0)	43 (10.0)	43 (10.0)	13 (13.5)	13 (13.5)	13 (13.5)	13 (13.5)
**To what extent do results of (inter)national studies and guidelines influence your usage of PSA testing?**	*Not at all*	1 (0.2)				5 (5.2)			
	*Weak*	5 (1.2)				5 (5.2)			
	*Moderate*	117 (27.1)				35 (36.5)			
	*Strong*	187 (43.3)				31 (32.3)			
	*Very strong*	54 (12.5)				6 (6.3)			
	*Missing replies*	68 (15.7)				14 (14.6)			

DEGAM, German Society of General Practice and Family Medicine; ERSPC, European Randomized Study of Screening for Prostate Cancer; GPs, general practitioners; PLCO, Prostate, Lung, Colorectal and Ovarian trial; PSA, prostate-specific antigen.

Results of (inter)national studies and guidelines appeared to have a stronger influence on the use of PSA testing for urologists than for GPs. Two-thirds of the urologists (55.8%, n=241) stated that study results and guidelines had a “very strong” or “strong” influence, compared with 38.6% of the GPs (n=37). Six urologists (1.4%) and 10 GPs (10.4%) answered that study results and guidelines had “no influence at all” or a “weak influence” on their use of PSA testing.

### Attitudes on PSA Testing

Urologists appeared more convinced about the PSA test as an early detection method compared with GPs (**Table 3**). Fewer GPs (61.5%, n=59) than urologists (87.1%, n=376) judged the early detection of PCa as “very important” or “important”. Most urologists (86.8%, n=375) judged the PSA test as “very meaningful” or “meaningful” compared with about half of the GPs (52.1%, n=50). Almost 80% of male urologists (79.9%, n=291) answered that they would undergo a PSA test themselves in the future (again), compared with 55.1% of male GPs (n=38). Almost two-thirds of the urologists (64.4%, n=278) thought that the reduction of PCa-related mortality by early detection based on PSA testing was “clearly proven” compared with one-fifth of the GPs (20.8%, n=20). Individual health services on PCa early detection were offered more frequently to patients in urological practices compared with general practices. Furthermore, individual health services in urological practices were more specialized (e.g., transrectal ultrasound examination of the prostate [73.9%, n=209] and the non-invasive NMP22^®^ BladderChek^®^ test for diagnosis and monitoring of bladder cancer [55.1%, n=156]), whereas laboratory tests (e.g., blood count [53.7%, n=22]) were offered most often in general practices (data not shown).

**Table 3:** Attitudes of urologists and general practitioners toward prostate-specific antigen testing [n (%)].

Question	Categories	Urologists (n=432)	GPs (n=96)
**How important do you think is the early detection of cancer in general?**	*Very unimportant*	2 (0.5)	2 (2.1)
	*Unimportant*	0 (0.0)	0 (0.0)
	*Undecided*	4 (0.9)	12 (12.5)
	*Important*	109 (25.2)	33 (34.4)
	*Very important*	271 (62.7)	36 (37.5)
	*Missing replies*	46 (10.6)	13 (13.5)
**How important do you think is the early detection of prostate cancer?**	*Very unimportant*	0 (0.0)	3 (3.1)
	*Unimportant*	3 (0.7)	8 (8.3)
	*Undecided*	7 (1.6)	13 (13.5)
	*Important*	107 (24.8)	28 (29.2)
	*Very important*	269 (62.3)	31 (32.3)
	*Missing replies*	46 (10.6)	13 (13.5)
**How do you judge the PSA test in general?**	*Not at all meaningful*	1 (0.2)	5 (5.2)
	*Not meaningful*	2 (0.5)	10 (10.4)
	*Neither/nor*	6 (1.4)	18 (18.8)
	*Meaningful*	176 (40.7)	34 (35.4)
	*Very meaningful*	199 (46.1)	16 (16.7)
	*Missing replies*	48 (11.1)	13 (13.5)
**Did you ever undergo a PSA test?** *Just men (n=364; n=69)*	*Yes*	269 (73.9)	37 (53.6)
	*No*	27 (7.4)	19 (27.5)
	*Does not (yet) apply*	25 (6.9)	5 (7.2)
	*Missing replies*	43 (11.8)	8 (11.6)
**Would you (again) undergo a PSA test in the future?** *Just men (n=364; n=69)*	*No, never*	3 (0.8)	5 (7.2)
	*No, rather not*	3 (0.8)	5 (7.2)
	*Undecided*	2 (0.5)	4 (5.8)
	*Yes, possibly*	14 (3.8)	7 (10.1)
	*Yes, in any case*	291 (79.9)	38 (55.1)
	*Does not apply*	8 (2.2)	2 (2.9)
	*Missing replies*	43 (11.8)	8 (11.6)
**Would you recommend a PSA test for early detection to a loved one (e.g. partner, father or brother)?** *Just women (n=68; n=27)*	*No, never*	0 (0.0)	5 (18.5)
	*No, rather not*	0 (0.0)	1 (3.7)
	*Undecided*	0 (0.0)	7 (25.9)
	*Yes, possibly*	9 (13.2)	1 (3.7)
	*Yes, in any case*	54 (79.4)	7 (25.9)
	*Does not apply*	0 (0.0)	6 (22.2)
	*Missing replies*	5 (7.4)	0 (0.0)
**Do you think that the reduction of prostate cancer-related mortality by early detection based on PSA testing is proven?**	*No, clearly not proven*	13 (3.0)	27 (28.1)
	*Undecided*	93 (21.5)	36 (37.5)
	*Yes, clearly proven*	278 (64.4)	20 (20.8)
	*Missing replies*	48 (11.1)	13 (13.5)
**Do you support the introduction of the PSA test as a statutory health insurance performance?**	*Yes*	244 (56.5)	41 (42.7)
	*No*	60 (13.9)	26 (27.1)
	*Unsure*	80 (18.5)	16 (16.7)
	*Missing replies*	48 (11.1)	13 (13.5)
**Does your practice offer individual health services (“IGel”) for early cancer detection?**	*Yes*	283 (65.5)	41 (42.7)
	*No*	79 (18.3)	41 (42.7)
	*Missing replies*	70 (16.2)	14 (14.6)

GPs, general practitioners; IGeL, “individuelle Gesundheitsleistung” or individual health service that must be paid by the patient; PSA, prostate-specific antigen.

### Usage of PSA Testing

In most practices, a standard procedure for PSA testing was available. If this standard procedure was available, it had been in place for 10 or more years in more than half of the cases (**Table 4**). Most physicians (e.g., instead of a medical assistant) were responsible for medical consultations on PSA testing (urologists: 87.7%, n=379; GPs: 88.5%, n=85), which was generally completed orally (urologists: 84.3%, n=364; GPs: 88.5%, n=85).

**Table 4:** Use of prostate-specific antigen testing among urologists and general practitioners [n (%)].

Variable	Categories	Urologists (n=432)	GPs (n=96)
**Is there a standard procedure regarding PSA testing (in your practice)?**	*Yes*	353 (81.7)	66 (68.8)
	*No*	43 (10.0)	27 (28.1)
	*Missing replies*	36 (8.3)	3 (3.1)
- **If yes, how old is this standard?** *(n=353; n=66)*	*≤3 years*	21 (5.9)	8 (12.1)
	*4-9 years*	99 (28.0)	22 (33.3)
	*≥10 years*	233 (66.0)	35 (53.0)
	*Missing replies*	79 (18.3)	31 (32.3)
**Who is responsible for the medical consultation on PSA testing (in your practice)? ^a^**	*Physician*	379 (87.7)	85 (88.5)
	*Medical assistant*	61 (14.1)	18 (18.8)
	*No specific person*	5 (1.2)	3 (3.1)
	*No one, PSA consultation is not performed*	5 (1.2)	1 (1.0)
	*Other*	0 (0.0)	0 (0.0)
	*Missing replies*	38 (8.8)	4 (4.2)
**How do you ask the patient if there is a wish to do a PSA test? ^a^**	*Not actively*	22 (5.1)	29 (30.2)
	*Orally*	354 (81.9)	58 (60.4)
	*Standardized written form*	48 (11.1)	1 (1.0)
	*Other*	5 (1.2)	2 (2.1)
	*Missing replies*	40 (9.3)	6 (6.3)
**How is the consultation on PSA testing done? ^a^**	*Orally*	364 (84.3)	85 (88.5)
	*Give away info material*	121 (28.0)	7 (7.3)
	*Info material in waiting room*	102 (23.6)	1 (1.0)
	*Other*	7 (1.6)	1 (1.0)
	*Missing replies*	40 (9.3)	6 (6.3)
**Did your own usage of the PSA test change in the last ten years?**	*Yes*	218 (50.5)	36 (37.5)
	*No*	146 (33.8)	46 (47.9)
	*Missing replies*	68 (15.7)	14 (14.6)
- **If yes, in which direction did your usage of the PSA test change? I perform a PSA test … than ten years ago.** *(n=218; n=36)*	*… much rarer …*	0 (0.0)	6 (16.7)
	*… rarer …*	103 (47.2)	13 (36.1)
	*… more frequent …*	108 (49.5)	13 (36.1)
	*… much more frequent …*	6 (2.8)	4 (11.1)
	*Missing replies*	1 (0.5)	0 (0.0)
**How many years of life expectancy does an asymptomatic patient need to have at least for you to recommend a PSA test?**	*Irrespective of life expectancy (meaning also for patients with life expectancy of <5 years)*	28 (6.5)	18 (18.8)
	*5-9 years*	67 (15.5)	11 (11.5)
	*10-14 years*	170 (39.4)	23 (24.0)
	*≥15 years*	17 (3.9)	11 (11.5)
	*Not at all*	0 (0.0)	25 (26.0)
	*Missing replies*	150 (34.7)	8 (8.3)
**Which proportion of men aged 45 years and older in your practice finally receives (at least) one PSA test (irrespective of where the test is performed)?**	*Almost none*	1 (0.2)	23 (24.0)
	*About one quarter*	37 (8.6)	30 (31.3)
	*Approximately half*	100 (23.1)	16 (16.7)
	*About three quarters*	136 (31.5)	13 (13.5)
	*Almost all*	107 (24.8)	6 (6.3)
	*Missing replies*	51 (11.8)	8 (8.3)
**Laboratory test**	*In own practice*	161 (37.3)	n/a
	*External laboratory*	99 (22.9)	n/a
	*Other*	23 (5.3)	n/a
	*Missing replies*	149 (34.5)	n/a

GPs, general practitioners; n/a, not applicable; PSA, prostate-specific antigen; ^a^, multiple responses possible.

Almost 40% of urologists (39.4%, n=170) indicated they would reserve a PSA test for an asymptomatic patient with at least 10–14 years of life expectancy, whereas GPs’ answers regarding the years of life expectancy were more heterogeneous. The percentage of men aged ≥45 years that received (at least) one PSA test was lower among GPs than urologists (categories “almost all” and “about three-quarters” in urologists: 56.3%, n=243; GPs: 19.8%, n=19), but the answers showed wide variation. Almost 40% of urologists indicated the laboratory test was conducted in their own practice (37.3%, n=161), compared with about 20% who noted this was performed in an external laboratory (22.9%, n=99).

Almost all urologists (89.8%, n=388) reported they would recommend a PSA test to an asymptomatic patient without risk factors compared with 55.2% of GPs (n=53) (**Table 5**). Reported intervals for retesting were also shorter among urologists compared with GPs.

**Table 5:** Case scenarios for prostate-specific antigen testing by urologists and general practitioners [n (%)].

Question	Categories	Urologists (n=432)	GPs (n=96)
**Case scenario 1: Imagine you see an asymptomatic patient without risk factors. Would you recommend him a PSA test at a certain age?**	*Yes*	388 (89.8)	53 (55.2)
	*No*	12 (2.8)	32 (33.3)
	*Cannot reply to that question*	5 (1.2)	4 (4.2)
	*Missing replies*	27 (6.3)	7 (7.3)
**Case scenario 2: Imagine you see a 45-year old patient with a life expectancy of at least 10 years who does not ask for an early detection examination based on PSA testing in your practice. Would you actively address a PSA test?**	*Yes*	367 (85.0)	31 (32.3)
	*No*	34 (7.9)	58 (60.4)
	*Missing replies*	31 (7.2)	7 (7.3)
**Case scenario 3: Imagine a 45-year old patient with a life expectancy of at least 10 years, having a PSA level of 1–2 ng/mL. Which interval would you recommend for a PSA test?**	*Interval every year or more often*	81 (18.8)	11 (11.5)
	*Interval every 2 years*	232 (53.7)	23 (24.0)
	*Interval every 3 years*	56 (13.0)	12 (12.5)
	*Interval every 4 years*	22 (5.1)	4 (4.2)
	*Interval less than every 4 years*	7 (1.6)	18 (18.8)
	*Not at all*	4 (0.9)	21 (21.9)
	*Missing replies*	30 (6.9)	7 (7.3)

GPs, general practitioners; PSA, prostate-specific antigen.

In general, urologists informed patients about PSA testing more frequently than GPs (**Table 6**). Men were more often informed about PSA testing in the context of early cancer detection or a positive family history compared with discomfort in the lower urinary tract by physicians in both specialties. Issues to inform the patient about were discussed by both urologists and GPs comparably often. Both groups reported they often discussed the benefit of early PCa detection and potential follow-up examinations if a test result was conspicuous, whereas the potential for anxiety during waiting for the test result was rarely mentioned to men. Although urologists conducted digital rectal examinations more frequently than GPs, early cancer detection examination was the main situation where a digital rectal examination was performed by physicians of both specialties.

**Table 6:** Information communication of prostate-specific antigen testing by urologists and general practitioners [n (%)].

Question	Categories	Urologists (n=432)						GPs (n=96)					
		*Never*	*Rarely*	*Sometimes*	*Often*	*Always*	*Missing replies*	*Never*	*Rarely*	*Sometimes*	*Often*	*Always*	*Missing replies*
**On which occasions do you inform your patient on PSA testing? In the context of …**	*… an early cancer detection*	3 (0.7)	1 (0.2)	2 (0.5)	59 (13.7)	324 (75.0)	43 (10.0)	8 (8.3)	5 (5.2)	10 (10.4)	29 (30.2)	38 (39.6)	6 (6.3)
	*… a positive family history*	2 (0.5)	0 (0.0)	4 (0.9)	26 (6.0)	357 (82.6)	43 (10.0)	5 (5.2)	5 (5.2)	12 (12.5)	25 (26.0)	43 (44.8)	6 (6.3)
	*… discomfort in the lower urinary tract*	7 (1.6)	21 (4.9)	63 (14.6)	160 (37.0)	138 (31.9)	43 (10.0)	14 (14.6)	13 (13.5)	18 (18.8)	27 (28.1)	18 (18.8)	6 (6.3)
**How often do you discuss the following aspects with your patients before performing a PSA test?**	*Benefit of PCa early detection*	3 (0.7)	2 (0.5)	10 (2.3)	81 (18.8)	286 (66.2)	50 (11.6)	2 (2.1)	1 (1.0)	4 (4.2)	40 (41.7)	41 (42.7)	8 (8.3)
	*Risks of PCa early detection (potential of overdiagnosis)*	4 (0.9)	17 (3.9)	47 (10.9)	112 (25.9)	202 (46.8)	50 (11.6)	5 (5.2)	8 (8.3)	9 (9.4)	29 (30.2)	37 (38.5)	8 (8.3)
	*State of the art*	14 (3.2)	51 (11.8)	112 (25.9)	123 (28.5)	81 (18.8)	51 (11.8)	15 (15.6)	13 (13.5)	21 (21.9)	24 (25.0)	15 (15.6)	8 (8.3)
	*Issue of false positives*	4 (0.9)	15 (3.5)	58 (13.4)	124 (28.7)	181 (41.9)	50 (11.6)	6 (6.3)	6 (6.3)	11 (11.5)	31 (32.3)	34 (35.4)	8 (8.3)
	*Potential anxiety during waiting on test result*	48 (11.1)	86 (19.9)	100 (23.1)	76 (17.6)	72 (16.7)	50 (11.6)	16 (16.7)	18 (18.8)	19 (19.8)	17 (17.7)	18 (18.8)	8 (8.3)
	*Potential follow-up examinations if the test result is conspicuous*	6 (1.4)	15 (3.5)	54 (12.5)	134 (30.3)	173 (25.9)	50 (11.6)	1 (1.0)	3 (3.1)	13 (13.5)	40 (41.7)	31 (32.3)	8 (8.3)
	*Adverse effects of the treatment*	14 (3.2)	43 (10.0)	82 (19.0)	131 (34.3)	112 (29.3)	50 (11.6)	7 (7.3)	12 (12.5)	17 (17.7)	29 (30.2)	23 (24.0)	8 (8.3)
**How often do you examine digito rectally in the following situations?**	*During an early cancer detection examination*	0 (0.0)	1 (0.2)	1 (0.2)	15 (3.5)	346 (80.1)	69 (16.0)	2 (2.1)	5 (5.2)	5 (5.2)	8 (8.3)	62 (64.6)	14 (14.6)
	*If there is blood in the patient ´s stool*	6 (1.4)	13 (3.0)	20 (4.6)	31 (7.2)	293 (67.8)	69 (16.0)	2 (2.1)	4 (4.2)	9 (9.4)	26 (27.1)	41 (42.7)	14 (14.6)
	*If the patient has a voiding disorder*	0 (0.0)	6 (1.7)	26 (6.0)	90 (20.8)	241 (55.8)	69 (16.0)	5 (5.2)	14 (14.6)	24 (25.0)	23 (24.0)	16 (16.7)	14 (14.6)
	*If the patient is asymptomatic*	34 (7.9)	40 (9.3)	94 (21.8)	103 (23.8)	91 (21.1)	70 (16.2)	47 (49.0)	25 (26.0)	9 (9.4)	1 (1.0)	(0.0)	14 (14.6)

GPs, general practitioners; PCa, prostate cancer; PSA, prostate-specific antigen.

With respect to handling an increased PSA level, 85.0% of urologists (n=367) said they would retest after having an asymptomatic patient with an increased PSA level, compared with half of the GPs (44.8%, n=43) (**Table 7**). GPs preferred to refer the patient to a urologist. Almost three-quarters of the urologists (61.3%, n=265) recommend a prostate biopsy for further verification, and 49.3% (n=213) recommend multiparametric prostate magnetic resonance imaging.

**Table 7:** Handling an increased prostate-specific antigen level by urologists and general practitioners [n (%)].

Question		Categories	Urologists (n=432)	GPs (n=96)
**Which further actions did you take the last time having an asymptomatic patient with an increased PSA level? Did you …**	*… check the PSA level within a certain interval?*	Yes	367 (85.0)	43 (44.8)
		No	2 (0.5)	40 (41.7)
		Missing replies	63 (14.6)	13 (13.5)
	*… directly refer the patient to a urologist?*	Yes	n/a	51 (53.1)
		No	n/a	32 (33.3)
		Missing replies	n/a	13 (13.5)
**Assuming you decided to check the PSA level again which, again, is conspicuous. How did you proceed with your last patient, having an increased PSA level again? Did you …**	*… repeat the test again?*	Yes	127 (29.4)	12 (12.5)
		No	242 (56.0)	70 (72.9)
		Missing replies	63 (14.6)	14 (14.6)
	*… directly refer the patient to a urologist?*	Yes	n/a	76 (79.2)
		No	n/a	8 (8.3)
		Missing replies	n/a	12 (12.5)
	*… recommend a prostate biopsy?*	Yes	265 (61.3)	n/a
		No	100 (23.1)	n/a
		Missing replies	67 (15.5)	n/a
	*… recommend a multiparametric prostate MRI?*	Yes	213 (49.3)	n/a
		No	154 (35.6)	n/a
		Missing replies	65 (15.0)	n/a

GPs, general practitioners; MRI, magnetic resonance imaging; n/a, not applicable; PSA, prostate-specific antigen.

## Discussion

This survey on attitudes toward and use of PSA testing among urologists and GPs in Germany showed that urologists were more convinced about this method of early PCa detection compared with GPs. In accordance with specialist-specific recommendations, GPs showed a more reserved approach toward PSA testing than urologists.

GPs in our study were less convinced about the reduction of PCa-related mortality by early detection based on PSA testing than GPs in a study conducted in Australia (23). In that study, three-quarters of the 149 GPs surveyed believed that PSA testing was at least “somewhat effective” in reducing PCa mortality in males with average risk. In contrast, about one-fifth of the GPs in our study supported this assumption. However, a US-based study showed that three-quarters of the primary care physicians surveyed strongly disagreed, disagreed, or were undecided as to whether the PSA test extended life (24). This may be due, in part, to the results of a systematic review, concluding that at best, PCa screening leads to a small reduction in disease-specific mortality over 10 years but has no effect on overall mortality (9). As large clinical trials have shown inconsistent results with respect to whether PSA testing leads to a reduction in PCa mortality, and because guidelines handle different recommendations, the observed disagreement among physicians is understandable (2–8). A survey among 305 primary care physicians in Sweden found the majority of physicians reported a less positive attitude toward PSA testing compared with the physicians in our survey (25). Roughly one-quarter of respondents in the Swedish survey considered the PSA test was a good test, one-third stated that the test provided good guidance, and almost half regarded it as a good compliment to palpation. In our study, almost 90% of urologists judged the PSA test as very meaningful or meaningful, compared with about 50% of GPs. When asked if they would (again) undergo a PSA test in the future, almost 80% of the male urologists in our study indicated they would, compared with 55% of the male GPs. In contrast, only 17% of the surveyed physicians in Sweden responded that they would definitely take a PSA test themselves (25). Regarding counselling before PSA testing, the percentage of GPs in our study that “always” or “often” discussed the implications in cases with a raised PSA level (74%, n=71) was comparable with the percentage reported for GPs in Northern Ireland (71%, n=199) (26). The fact that urologists more frequently informed patients about PSA testing than GPs may be attributable to their belief in the PSA test, as belief in the efficacy of PSA screening is associated with recommendation for the test (27). Results of interviews among GPs in Australia and the United Kingdom suggested that GPs’ primary communication goals (encourage asymptomatic men to either have a PSA test, not test, or to support men to make their own decision) were a central component of consultations about PCa screening (28).

Several studies have shown that there is a wide variation in PSA testing practices (18, 23, 29). In our study, answers to the question concerning the proportion of men aged ≥45 years that received (at least) one PSA test varied widely, whereas the frequency of ordering PSA tests among GPs in Sweden showed moderate variation (25). Compared with primary care physicians in the US, physicians in our study were more proactive in recommending a PSA test (24). One-quarter of physicians in the US study routinely offered and recommended a PSA test to all asymptomatic male patients of screening age, regardless of whether the patient asked about the test. In contrast, almost 90% of the urologists and 55% of the GPs in our study would recommend a PSA test to an asymptomatic patient without risk factors at a certain age. The study from Northern Ireland found that 80% of the responding GPs tested all men with lower urinary tract symptoms, and 65% tested men with a positive family history of prostatic carcinoma (26). In the context of discomfort in the lower urinary tract, almost 70% of urologists and almost half of GPs in our study “always” or “often” informed their patient about PSA testing. In cases with a positive family history, information about PSA testing was provided by almost 90% of urologists and about 70% of GPs in our study. In a survey involving 325 GPs in Denmark, 28% of the GPs measured PSA in patients with lower urinary tract symptoms (30). In our study, almost 70% of the participating urologists worked in the outpatient setting, increasing the comparability of the results to the participating GPs. Although no big differences were expected between urologists working in the outpatient setting and urologists working in the hospital, small differences were conceivable. Urologists working in the outpatient setting might for example handle a more practically orientated, pragmatic or time-saving approach, while urologists working in hospitals might take more time to inform patients or have better knowledge of guidelines/study results, possibly in dependency on status of the hospital (academic medical center vs. local hospital). In addition to urologists being more convinced about the PSA test than GPs, another reason that may explain differences between the specialist groups is that almost 40% of the urologists had a laboratory in their own practice. It is therefore conceivable that monetary factors may influence the use of PSA tests among urologists. Furthermore, differences in healthcare systems across countries may also explain the observed results. The finding that many GPs in England retested men with a raised PSA level rather than making an immediate referral was supported by our results, as about 45% of the GPs in our study rechecked the PSA level within a certain interval (31). The study from Denmark reported that 52% of the surveyed GPs would refer an asymptomatic patient with an elevated PSA level (30). A systematic review concluded that follow-up after a normal or raised PSA test by GPs and non-urologic hospitalists varied greatly and did not appear to be in accordance with practice guidelines (29). Recommendations on PSA testing influence the daily practice of PSA testing among physicians (32). For example, 56% of the urologists in our study stated that the results of (inter)national studies and guidelines “very strongly” or “strongly” influenced their use of PSA testing compared with around 40% of the GPs. In another survey among urologists in Germany, 93% of the responding urologists reported they used the German S3 guideline in daily practice, and 95% considered the strong recommendations of the guideline as the treatment standard (33). Barriers for guideline adherence were among others patient-related factors, suggesting that current guidelines do not always adequately incorporate patient preferences, needs and abilities (34). Further barriers were lack of time, patient pressure, and guidelines being too long, rigid or unclear (35).

Major strengths of this study were the inclusion of physicians of different specialties, the inclusion of urologists working in both clinics and outpatient settings, and the nationwide coverage of urologists. Although we were not able to objectively measure the usage of PSA testing, this survey gives us an impression about the real-world daily practice of attitudes on and usage of PSA testing among physicians. Urologists were compared with participating GPs from a single federal state, whereby about 13% of the participating urologists worked in this state. This might have introduced limitations in terms of selection bias and problems with the representativeness of our results. Further, a higher number of participating GPs would have been helpful. Possible explanations for the low response proportions among both specialist groups include lack of time, lack of reimbursement, lack of interest, and the volume of online questionnaires that physicians receive. It is conceivable that the physicians who participated in this survey had a special interest in this topic or research, which might have led to an underestimation of the results.

To enable men to access the best available urological healthcare, further research on the net usefulness of PSA testing and optimal implementation of the test in clinical practice is essential. As other authors have suggested, there is a need for further high-level professional discussions about the primary goals of physicians when communicating about PSA screening (28). Another important step is raising awareness about early detection of PCa among the male population, as suggested in the EU Cancer Plan (14). In the interests of all patients and physicians, it would be helpful to achieve an internationally consistent approach toward PSA testing.

## Conclusion

GPs are more skeptical about PSA testing than urologists. GPs therefore use the PSA test less frequently compared with urologists, although the preselected patient population in the urological setting must be considered. Our findings are consistent with the specialist-specific recommendations on early detection of PCa. To further improve urooncological healthcare, it would be helpful to achieve a consistent approach toward PSA testing.

## Data Availability Statement

The original contributions presented in the study are included in the article. Further inquiries can be directed to the corresponding author.

## Author Contributions

SK, VJ, and AW were responsible for the study design and literature search. All authors developed the questionnaire together. MHF and AW were responsible for conducting the questionnaire. SK performed the descriptive analysis and the data management. SK and AW interpreted the data. SK drafted the manuscript, and VJ, MF, and AW revised the text. All authors contributed to the article and approved the submitted version.

## Funding

This study was funded by the Research Pool of the Carl von Ossietzky University Oldenburg, Germany (grant number: FP 2016-I_Kappen-Winter).

## Conflict of Interest

MHF reports personal fees from DAK Gesundheit outside the submitted work.

The remaining authors declare that the research was conducted in the absence of any commercial or financial relationships that could be construed as a potential conflict of interest.

## Abbreviations

DEGAM, German Society of General Practice and Family Medicine; ERSPC, European Randomized Study of Screening for Prostate Cancer; GP, General practitioner; PCa, Prostate cancer; PLCO, Prostate, Lung, Colorectal and Ovarian Cancer Screening Trial; PSA, Prostate-specific antigen.
